# Obesity paradox in individuals with type 1 diabetes

**DOI:** 10.3389/fcdhc.2025.1670312

**Published:** 2025-10-01

**Authors:** Hidetaka Hamasaki

**Affiliations:** Japanese Academy of Health and Practice, Tokyo, Japan

**Keywords:** obesity, type 1 diabetes, body mass index, body composition, skeletal muscle, fat, mortality

## Abstract

The obesity paradox describes a counterintuitive phenomenon where overweight or mildly obese individuals with chronic diseases show better survival compared to those with normal weight. While this paradox has been reported in conditions such as heart failure and type 2 diabetes, its presence in type 1 diabetes (T1D) remains uncertain. This mini review summarizes current evidence from large cohort studies and a meta-analysis examining the association between body mass index (BMI) and clinical outcomes in individuals with T1D. Most findings do not support a protective effect of higher BMI; instead, both underweight and obesity are associated with increased risks of cardiovascular events and all-cause mortality. Notably, some evidence suggests that individuals with advanced diabetic nephropathy or chronic kidney disease (CKD) may show the lowest mortality at mildly elevated BMI levels. However, these observations may reflect the limitations of using BMI alone to evaluate obesity. Given that individuals with T1D often have reduced skeletal muscle mass, and that those with advanced diabetic complications or comorbidities such as CKD or cancer may develop cachexia, body composition analysis is essential. Accurate assessment of fat mass, muscle mass, bone mass, and water content is critical for understanding obesity-related risks. Future research should integrate body composition metrics to improve risk stratification in T1D.

## 1 Introduction

The obesity paradox refers to a phenomenon in which, contrary to the general understanding that obesity increases the risk of cardiovascular (CV) disease and mortality, individuals with preexisting conditions such as heart failure (1, 2), chronic kidney disease (CKD) (3), or type 2 diabetes (T2D) (4) exhibit higher survival rates when classified as “overweight to mildly obese” (approximately body mass index (BMI) 25 to 30 kg/m²) compared to those with normal or low body weight. Several hypotheses have been proposed to explain this paradox, including (a) bias resulting from the tendency of such patients to become underweight and malnourished due to the effects of the disease itself (5), (b) confounding factors such as smoking (6), (c) limitations of BMI in capturing differences in muscle mass and fat distribution (7–9), (d) reduced sympathetic activation observed in conditions such as obesity and heart failure (10), and (e) increased energy expenditure and muscle catabolism caused by inflammation and elevated metabolic demands in chronic illnesses, with greater fat and muscle reserves potentially acting as protective nutritional buffers (11). However, causal validation through well designed studies including randomized controlled trials remains limited, and it is still unclear whether this paradox represents a universal phenomenon.

The obesity paradox in patients with diabetes remains a subject of ongoing debate. However, emerging evidence suggests that observed outcomes vary depending on patient demographics and the clinical characteristics of the disease. One cohort study reported that a BMI in the overweight range (25 to 30 kg/m²) was associated with the lowest risk of all-cause mortality and CV events, indicating a more favorable prognosis (12). In contrast, another study demonstrated that the obesity paradox was not observed among non-smokers (13). Furthermore, an analytical review concluded that the obesity paradox is largely driven by reverse causation and confounding factors, particularly smoking and severe comorbid conditions, and that obesity is in fact associated with increased mortality (14). These findings raise the question of whether the obesity paradox is also present in individuals with type 1 diabetes (T1D). Although both T1D and T2D are characterized by chronic hyperglycemia, they are fundamentally distinct in terms of their underlying pathophysiological mechanisms. In recent years, the prevalence of obesity has increased among individuals with T1D (15). Nevertheless, obesity remains more prevalent in those with T2D (16), and while it plays a major role in the pathogenesis of T2D, it is not directly implicated in the development of T1D (17). In this mini review, the author summarizes previous clinical studies to evaluate whether the obesity paradox exists in individuals with T1D. The author further discusses its potential underlying mechanisms and the clinical implications that should be considered in patient care.

## 2 Current evidence

The study by Edqvist et al. (18) evaluated the relationship between BMI and adverse outcomes in individuals with T1D, utilizing data from the Swedish National Diabetes Register. A total of 26,125 patients without established CV disease were followed for a median duration of 10.9 years. For all-cause mortality, the overall incidence rate (per 1,000 person years) was 3.92 (95% confidence interval (CI), 3.68 to 4.16). Among patients with normal BMI (18.5 to less than 25 kg/m²), the rate was 3.83, increasing to 3.95 in the overweight group (25 to less than 30 kg/m²), and further to 4.39 in the obese group (30 kg/m² or higher). CV mortality followed a similar trend. The overall incidence was 1.30 (95% CI, 1.16 to 1.44); within the normal BMI group, the rate was 1.18, rising to 1.42 in the overweight group and to 1.67 in the obese group. For major CV events, the overall incidence was 5.69 (95% CI, 5.40 to 5.99). Individuals with normal BMI had a rate of 4.99, compared with 6.58 and 6.94 in the overweight and obese groups, respectively. Regarding hospitalization for heart failure, the overall incidence was 2.22 (95% CI, 2.04 to 2.41), with corresponding rates of 1.84 in the normal weight group, 2.72 in the overweight group, and 2.85 in the obese group. These findings demonstrate a consistent increase in adverse outcomes with higher BMI, thereby challenging the concept of an obesity paradox in individuals with T1D. Rather than conferring protective effects, higher BMI was independently associated with elevated risks of CV events, heart failure, and mortality. The authors emphasized that excess adiposity in T1D negatively affects long term outcomes and that weight management should be prioritized as an important clinical target.

A longitudinal cohort study from the Finnish Diabetic Nephropathy study examined the association between BMI and mortality in individuals with T1D (19). Among 6,957 adults initially identified, a final sample of 5,836 individuals was included in the mortality analysis after excluding those with unknown renal status, age at diagnosis greater than 40 years, and inadequate follow-up. Over a median follow-up of 13.7 years, 876 deaths occurred. Using World Health Organization BMI categories, underweight individuals exhibited a markedly increased risk of mortality compared with those of normal weight (hazard ratio (HR) = 4.26; 95% CI, 2.84 to 6.39), whereas obese individuals had a 25% higher mortality risk (HR = 1.25; 95% CI, 1.01 to 1.54), and overweight individuals demonstrated a modest reduction in mortality risk (HR = 0.86; 95% CI, 0.74 to 0.99). A reverse J-shaped relationship was observed between BMI and all-cause mortality, while a U-shaped association was seen for CV mortality. The BMI associated with the lowest risk of mortality was 24.3 kg/m² for all-cause mortality and 24.8 kg/m² for CV mortality. These nonlinear associations remained significant after adjustment for diabetic nephropathy (DN) status, chronic kidney disease (CKD) status, systolic blood pressure, use of antihypertensive agents, and hemoglobin A1c (HbA1c). The relationship between BMI and mortality differed significantly by DN status, but not by CKD status as defined by estimated glomerular filtration rate. Among individuals without DN or CKD, the nadir BMI associated with the lowest mortality was 24.0 kg/m², whereas in those with DN or CKD, the nadirs were 26.1 and 25.9 kg/m², respectively. Moreover, the association between BMI and mortality varied significantly by sex and age at diabetes onset, but not by chronological age, HbA1c, or smoking history. In summary, this study provides evidence that both low and high BMI are associated with increased mortality in individuals with T1D, with optimal survival observed at BMI levels within the normal to mildly elevated range. Although the study does not provide a definitive conclusion regarding the presence or absence of the obesity paradox, it underscores the importance of diabetes-related complications, such as renal function and DN, along with demographic factors, in discussions of BMI-related mortality.

An observational study investigated whether obesity and diabetes influence short-term survival following out-of-hospital cardiac arrest (20). A total of 55,483 adult cases were identified in the Swedish Registry of Cardiopulmonary Resuscitation between January 1, 2010, and December 31, 2020. Among these, 12,700 individuals (22.9%) had a history of obesity, diabetes, or both conditions. Individuals with obesity alone (n = 1,516) were younger, with a mean age of 62.0 years. Patients with T1D (n = 432) had a mean age of 64.7 years, whereas those with T2D (n = 9,026) had the highest mean age, at 75.1 years. The prevalence of CV comorbidities such as hypertension and heart failure was notably higher in groups with diabetes or obesity compared to those without these conditions. Thirty-day survival rates were 12.7% in the reference group without obesity or diabetes (n = 43,467), 9.6% in the obesity alone group, 10.6% in the T1D group, 7.3% in the T2D group, and 6.9% in the group with both obesity and diabetes (n = 1,762). After adjustment for age and sex, the odds ratio (OR) for thirty-day survival was 0.69 (95% CI, 0.57 to 0.82) for individuals with obesity alone, 0.78 (95% CI, 0.56 to 1.05) for those with T1D, 0.65 (95% CI, 0.59 to 0.71) for those with T2D, and 0.55 (95% CI, 0.45 to 0.66) for individuals with both obesity and diabetes. Further adjustment for location of arrest, time to resuscitation, and initial cardiac rhythm did not substantially alter these associations. These findings do not support the presence of an obesity paradox in individuals with diabetes. The presence of obesity did not improve survival, and among individuals with both obesity and diabetes, survival was significantly reduced. However, whether an obesity paradox exists in individuals with T1D remains unclear, as the analysis did not separately assess individuals with both obesity and T1D or those with both obesity and T2D.

Although only three studies were included in the analysis, Jung and colleagues (21) conducted a systematic review and meta-analysis to investigate the risk of all-cause mortality across different BMI categories among individuals with T1D. The study by Dahlström (19), mentioned above, was one of the three studies included. This systematic review and meta-analysis incorporated data from 23,047 patients drawn from three longitudinal cohort studies (19, 22, 23). BMI categories were defined according to the World Health Organization classification, with the reference group consisting of individuals with normal weight, defined as a BMI between 18.5 and less than 25 kg/m². The pooled HR for the underweight group (BMI less than 18.5 kg/m²) was 3.38 (95% CI, 1.67 to 6.85). In contrast, individuals in the overweight category (BMI 25 to 30 kg/m²) had an HR of 0.90 (95% CI, 0.66 to 1.22), indicating no significant difference in mortality. For individuals classified as obese (BMI 30 kg/m² or higher), the pooled HR was 1.36 (95% CI, 0.86 to 2.15). Heterogeneity across the three studies was moderate to high for all non-reference categories; however, the directional trends were consistent. In summary, the authors found no evidence supporting the existence of the so-called obesity paradox in individuals with T1D. However, the findings suggest a U-shaped relationship between BMI and mortality, with the lowest risk observed among individuals in the normal BMI range. Both underweight and, to a lesser extent, obesity were associated with increased mortality risk. These results show the importance of maintaining an appropriate weight range in the management of T1D and highlight the need for future research on the role of body composition and metabolic health.

**Table 1** summarizes the results of the aforementioned studies.

**Table 1:** Clinical studies on the obesity paradox in individuals with type 1 diabetes.

Author, Year	Study design	Participants	Main findings
Edqvist et al., 2019 (18)	Prospective cohort study	26,125 individuals with T1D Age: 33.3 ± 13.0 years; 44.5% women BMI 24.8 ± 3.6 kg/m^2^ (baseline)	Higher BMI was associated with an increased risk of heart failure, cardiovascular events, and mortality in men. No obesity paradox was observed after excluding factors related to reverse causality.
Dahlström et al., 2019 (19)	Prospective cohort study	5,836 individuals with T1D Age: 38.9 years; 51.3% men BMI: 25.0 ± 3.6 kg/m^2^ (in 1990s), 25.2 ± 3.7 kg/m^2^ (in 2000s), 26.4 ± 4.6 kg/m^2^ (in 2010s)	A reverse J-shaped association between BMI and mortality was observed in patients with DN. A higher BMI (approximately 26 kg/m²) appeared to be protective in those with DN or CKD, suggesting a possible obesity paradox.
Hjalmarsson et al., 2023 (20)	Registry-based observational cohort study	55,483 OHCA cases; 1,516 with obesity alone, 432 with T1D, 9,026 with T2D, 1,762 with obesity and diabetes Age: No description BMI: No description	Obesity, either alone or in combination with diabetes, was associated with reduced 30-day survival after OHCA. No obesity paradox was observed.
Jung et al., 2023 (21)	Systematic review and meta-analysis	3 prospective studies, 23,407 adults with T1D; 50.8–55.0% men Age 28–39.5 years BMI: No description	Being underweight was significantly associated with increased mortality, whereas no significant difference in mortality was observed among individuals who were overweight or obese.

Some values are presented as mean ± standard deviation. T1D, type 1 diabetes; BMI, body mass index; DN, diabetic nephropathy; CKD, chronic kidney disease; OHCA, out-of-hospital cardiac arrest; T2D, type 2 diabetes.

## 3 Discussion

Recent investigations have explored the validity of the obesity paradox in individuals with T1D. Large prospective cohort studies, including those conducted in Sweden and Finland, have consistently demonstrated that a higher BMI is not associated with improved survival in this population. Instead, a nonlinear relationship has been observed, whereby both low and high BMI are linked to increased risks of all-cause and CV mortality. Collectively, these findings suggest that the optimal BMI range for favorable long-term outcomes lies within the normal to mildly elevated category. Notably, the Swedish National Diabetes Register study reported a graded increase in adverse CV events and mortality with increasing adiposity, underscoring the detrimental impact of excess body weight even in individuals without baseline CV disease. Similarly, findings from the Finnish Diabetic Nephropathy cohort indicated that the relationship between BMI and mortality is further modified by diabetes-related complications, such as nephropathy and impaired kidney function. In the context of acute events, such as out-of-hospital cardiac arrest, national registry data likewise failed to demonstrate any survival advantage associated with obesity or its coexistence with diabetes. These results further challenge the hypothesis that excess body weight confers cardiometabolic resilience in individuals with T1D. Complementing these findings, a recent systematic review and meta-analysis synthesizing data from multiple cohorts concluded that the obesity paradox is not supported in this population. However, maintaining a healthy body composition appears to be a critical goal for mitigating long-term mortality risk. Future research should investigate the roles of fat distribution, skeletal muscle mass, and overall metabolic health to refine individualized management strategies.

The number of individuals with T1D who are also obese is increasing, and debate continues regarding both the impact of obesity on the pathophysiology of T1D and the reasons why those with T1D are prone to obesity. For example, individuals with T1D who experience frequent hypoglycemic episodes may overconsume high-carbohydrate foods, and it has been reported that an additional intake of only 15 g of carbohydrates per day can result in approximately 2.7 kg of annual weight gain. Moreover, endocrine alterations such as abnormal glucagon or amylin secretion in the absence of endogenous insulin, together with intensive insulin therapy, have been shown to promote weight gain (24). Although these observations do not indicate the presence of an obesity paradox, a noteworthy case report from Japan described fulminant T1D with severe obesity and positive anti-GAD antibodies, in which pronounced obesity appeared to enhance ketone body production and aggravate acidosis (25). Thus, while it remains unclear whether obesity precedes the onset of T1D or develops subsequently, progression of obesity is likely to have adverse effects on health in the context of T1D. At the same time, as previously noted, underweight individuals with T1D have a 3.4-fold higher mortality risk compared with those of normal weight, whereas those who are overweight or obese do not show a significant increase in mortality risk (21). Furthermore, although based on a small cross-sectional analysis, an investigation of the relationship between glycemic indices monitored by continuous glucose monitoring, body weight, and BMI in T1D found that both weight and BMI were positively associated with improved glycemic control (26). From the perspective of the obesity paradox, these findings suggest that maintaining a certain level of body weight and BMI may help stabilize glycemic variability and support better overall health. Nevertheless, the optimal weight and BMI remain undefined in the current literature.

In individuals with T1D, skeletal muscle fat content has been reported to be higher than in those with T2D. Conversely, individuals with T2D exhibit more pronounced visceral fat accumulation, which is strongly associated with increased insulin resistance (27). Importantly, even T1D patients with a normal or mildly elevated BMI demonstrate significantly higher CV risk scores when visceral fat levels are elevated. This finding suggests that “hidden visceral obesity,” which is not adequately captured by conventional metrics such as BMI or waist circumference, may contribute to the underestimation of CV risk in this population (28). Ectopic fat depots have been shown to secrete bioactive substances that influence insulin resistance, glucose and lipid metabolism, coagulation, and inflammatory pathways, thereby heightening the risk of CV disease and atherosclerosis (29, 30).

On the other hand, accumulating evidence indicates that greater muscle mass is associated with lower mortality (31–33). A study by Wei et al. (34) showed that a higher appendicular skeletal muscle mass to visceral fat area ratio, which reflects a relatively greater muscle mass compared to visceral adiposity, is associated with lower CV and cancer mortality in individuals with diabetes. Skeletal muscle mass tends to decline in individuals with diabetes due to multiple mechanisms, including impaired muscle protein synthesis resulting from insulin deficiency, activation of proteolytic pathways, increased protein breakdown driven by oxidative stress and inflammation, mitochondrial dysfunction, and reduced levels of insulin like growth factor 1 (35). Individuals with T1D, in particular, have been shown to exhibit decreased skeletal muscle mass (36, 37), which represents a significant health concern in addition to increases in fat mass. Adults with long-term T1D (more than 20 years) treated with insulin therapy exhibited higher adiposity and lower lean mass compared with healthy individuals (38). If T1D is regarded as one of the autoimmune diseases, the impact of autoimmunity on skeletal muscle mass must also be considered. Autoimmune diseases exert profound effects on skeletal muscle, primarily through chronic inflammation that drives sarcopenia. Proinflammatory cytokines such as interleukin-1β, interleukin-6, and tumor necrosis factor-α activate catabolic pathways including NF-κB and p38 MAPK, leading to proteolysis via ubiquitin ligases such as atrogin-1 and MuRF-1 (39). In rheumatoid arthritis, the prevalence of sarcopenia ranges from 10% to 45% and is associated with bone loss, frailty, and CV risk. In T1D, hyperglycemia promotes intramyocellular lipid accumulation, advanced glycation end-product deposition, and mitochondrial dysfunction, all of which impair muscle quality. These mechanisms highlight that autoimmunity accelerates muscle atrophy (39). Although obesity has been associated with a 34% lower risk of sarcopenia in older adults (OR = 0.66; 95% CI, 0.48 to 0.91), this association appears to depend on the preservation of muscle mass and strength (40). These observations emphasize the limitations of evaluating obesity and its health consequences solely through traditional metrics such as body weight, BMI, or waist to hip ratio, as well as measurements of visceral or total body fat.

In the author’s view, the most significant limitation of the obesity paradox lies in the way obesity is evaluated. Obesity should be evaluated not solely by body weight or BMI, but rather by body composition, which encompasses the relative proportions of muscle mass, fat mass, and body water. In the study by Dahlström and colleagues (19), individuals with T1D who had advanced DN or CKD exhibited the lowest mortality at a BMI of approximately 26, suggesting the potential presence of the obesity paradox. This suggests that the obesity paradox may manifest particularly in patients with diabetic complications.

The number of individuals with T1D who develop CKD is increasing, and there are reports indicating that, after adjusting for age, CKD is more prevalent among individuals with T1D than among those with T2D (41, 42). Diabetic complications can have a significant impact on the prognosis of diabetes patients. Individuals with diabetic neuropathy, one of the major complications of diabetes, often experience reductions in skeletal muscle mass (43, 44), while those with advanced CKD commonly develop fluid imbalances, deteriorating nutritional status, and muscle wasting, frequently presenting as cachexia or protein energy wasting (45). These pathophysiological changes may compromise the validity of using BMI alone to assess obesity. In addition, bone mass, which constitutes approximately 14% of total body weight, represents another critical factor (46). Bone mass declines with age, and this decline has been reported to progress more rapidly in individuals with obesity (47). Fat free mass and muscle strength are closely associated with bone mass, and visceral adiposity has been suggested to exert a detrimental effect on skeletal integrity (48).

Therefore, in order to accurately assess obesity and determine the presence or absence of the obesity paradox, it is essential to precisely quantify body composition components that contribute to body weight and BMI, including muscle mass, fat mass, bone mass, and body water. Furthermore, careful consideration must be given to factors such as age and disease status, which influence each of these components. For instance, in patients with cancer, nutritional and metabolic disturbances induced by malignancy can lead to pathological loss of muscle mass, resulting in a condition known as cancer cachexia (49). In such cases, evaluating obesity based solely on body weight or BMI becomes clearly inadequate. In the context of type 1 diabetes, which is the focus of this review, the presence of complications such as DN and CKD plays a critical role in the interpretation of BMI and its association with clinical outcomes.

**Figure 1:**
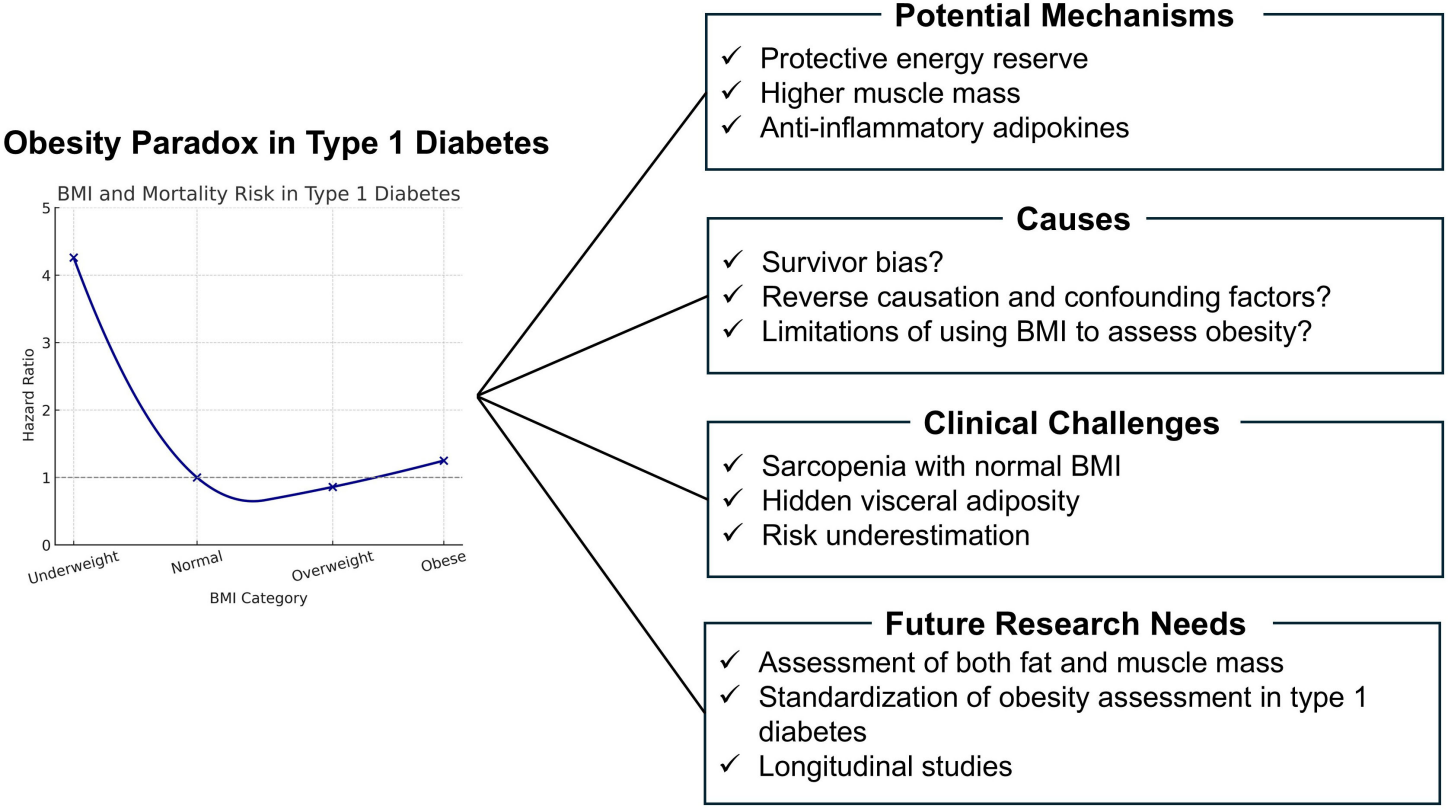
Obesity paradox in type 1 diabetes: Potential mechanisms, challenges, and future directions.

In conclusion, the current evidence does not support the presence of an obesity paradox in individuals with T1D (**Figure 1**). This mini review includes only three cohort studies and a single meta-analysis, which may limit the generalizability of the findings regarding the obesity paradox in T1D. The small number of included studies represents a major limitation. However, one study has reported that among those with advanced DN and CKD, individuals classified as overweight have the lowest mortality. Although the limited number of studies precludes definitive conclusions, this observation likely reflects the limitations of assessing obesity using BMI alone. In recent years, the average BMI among individuals with T1D has been increasing, highlighting the need for accurate evaluation of obesity. It is essential to assess body composition precisely, including muscle mass, fat mass, visceral fat, bone mass, and body water, rather than relying on body weight or BMI alone. Particular attention should be paid to imbalances, such as reduced muscle or bone mass accompanied by increased fat mass. Moreover, the evaluation must account for comorbid conditions, including diabetic complications and cancer. Continued improvement in measurement methodologies and further research are warranted.

## References

[1] Alebna PL Mehta A Yehya A daSilva-deAbreu A Lavie CJ Carbone S. Update on obesity, the obesity paradox, and obesity management in heart failure. Prog. Cardiovasc. Dis. (2024) 82:34–42. doi: 10.1016/j.pcad.2024.01.003, PMID: 38199320

[2] Merkel ED Behon A Masszi R. Obesity paradox in patients with reduced ejection fraction eligible for device implantation: an observational study. ESC Heart Fail. (2024) 11:3616–25. doi: 10.1002/ehf2.14961, PMID: 39031161 PMC11631315

[3] Naderi N Kleine CE Park C Hsiung JT Nguyen DV Ku E. Obesity paradox in advanced kidney disease: from bedside to the bench. Prog. Cardiovasc. Dis. (2018) 61:168–81. doi: 10.1016/j.pcad.2018.07.001, PMID: 29981348 PMC6131022

[4] Han SJ Boyko EJ. The evidence for an obesity paradox in type 2 diabetes mellitus. Diabetes Metab. J. (2018) 42:179–87. doi: 10.4093/dmj.2018.0055, PMID: 29885111 PMC6015958

[5] Preston SH Stokes A. Obesity paradox: conditioning on disease enhances biases in estimating the mortality risks of obesity. Epidemiology. (2014) 25:454–61. doi: 10.1097/EDE.0000000000000075, PMID: 24608666 PMC3984024

[6] Badrick E Sperrin M Buchan IE Renehan AG. Obesity paradox and mortality in adults with and without incident type 2 diabetes: a matched population level cohort study. BMJ Open Diabetes Res. Care. (2017) 5:e000369. doi: 10.1136/bmjdrc-2016-000369, PMID: 28321314 PMC5353321

[7] Hainer V Aldhoon Hainerová I. Obesity paradox does exist. Diabetes Care. (2013) 36 Suppl 2:S276–81. doi: 10.2337/dcS13-2023, PMID: 23882059 PMC3920805

[8] Bozorgmanesh M Arshi B Sheikholeslami F Azizi F Hadaegh F. No obesity paradox BMI incapable of adequately capturing the relation of obesity with all cause mortality an inception diabetes cohort study. Int. J. Endocrinol. (2014) 2014:282089. doi: 10.1155/2014/282089, PMID: 25180034 PMC4142289

[9] Donini LM Pinto A Giusti AM Lenzi A Poggiogalle E. Obesity or BMI paradox beneath the tip of the iceberg. Front. Nutr. (2020) 7:53. doi: 10.3389/fnut.2020.00053, PMID: 32457915 PMC7221058

[10] Farré N Aranyó J Enjuanes C Verdú JM Molina L Domingo M. Differences in neurohormonal activity partially explain the obesity paradox in patients with heart failure the role of sympathetic activation. Int. J. Cardiol. (2015) 181:120–6. doi: 10.1016/j.ijcard.2014.12.025, PMID: 25497534

[11] Webster JM Kempen LJAP Hardy RS Langen RCJ. Inflammation and skeletal muscle wasting during cachexia. Front. Physiol. (2020) 11:597675. doi: 10.3389/fphys.2020.597675, PMID: 33329046 PMC7710765

[12] Costanzo P Cleland JG Pellicori P Clark AL Hepburn D Kilpatrick ES. The obesity paradox in type 2 diabetes mellitus relationship of body mass index to prognosis a cohort study. Ann. Intern. Med. (2015) 162:610–8. doi: 10.7326/M14-1551, PMID: 25938991

[13] Tobias DK Pan A Jackson CL O’Reilly EJ Ding EL Willett WC. Body mass index and mortality among adults with incident type 2 diabetes. N Engl. J. Med. (2014) 370:233–44. doi: 10.1056/NEJMoa1304501, PMID: 24428469 PMC3966911

[14] Tobias DK Manson JE. The obesity paradox in type 2 diabetes and mortality. Am. J. Lifestyle Med. (2016) 12:244–51. doi: 10.1177/1559827616650415, PMID: 30202394 PMC6124964

[15] Kueh MTW Chew NWS Al Ozairi E Le Roux CW. The emergence of obesity in type 1 diabetes. Int. J. Obes. (Lond). (2024) 48:289–301. doi: 10.1038/s41366-023-01429-8, PMID: 38092958 PMC10896727

[16] Xu G Liu B Sun Y Snetselaar LG Hu FB Bao W. Prevalence of diagnosed type 1 and type 2 diabetes among US adults in 2016 and 2017 population based study. BMJ. (2018) 362:k1497. doi: 10.1136/bmj.k1497, PMID: 30181166 PMC6122253

[17] Abbasi A Juszczyk D van Jaarsveld CHM Gulliford MC. Body mass index and incident type 1 and type 2 diabetes in children and young adults: A retrospective cohort study. J. Endocr. Soc. (2017) 1:524–37. doi: 10.1210/js.2017-00044, PMID: 29264507 PMC5686575

[18] Edqvist J Rawshani A Adiels M Björnson E Gudbjörnsdottir S Svensson AM. BMI, mortality, and cardiovascular outcomes in type 1 diabetes findings against an obesity paradox. Diabetes Care. (2019) 42:1297–304. doi: 10.2337/dc18-1446, PMID: 31048408

[19] Dahlström EH Sandholm N Forsblom CM Thorn LM Jansson FJ Harjutsalo V. Body mass index and mortality in individuals with type 1 diabetes. J. Clin. Endocrinol. Metab. (2019) 104:5195–204. doi: 10.1210/jc.2019-00042, PMID: 31034018

[20] Hjalmarsson A Rawshani A Råmunddal T Nordberg P Svensson L Djärv T. No obesity paradox in out of hospital cardiac arrest data from the Swedish registry of cardiopulmonary resuscitation. Resusc Plus. (2023) 15:100446. doi: 10.1016/j.resplu.2023.100446, PMID: 37601410 PMC10432953

[21] Jung HN Kim S Jung CH Cho YK. Association between body mass index and mortality in type 1 diabetes mellitus a systematic review and meta analysis. J. Obes. Metab. Syndr. (2023) 32:151–62. doi: 10.7570/jomes22061, PMID: 37280725 PMC10327680

[22] Conway B Miller RG Costacou T Fried L Dorman JS Orchard TJ. Adiposity and mortality in type 1 diabetes. Int. J. Obes. (Lond). (2009) 33:796–805. doi: 10.1038/ijo.2009.75, PMID: 19451912 PMC2755198

[23] Vestberg D Rosengren A Eeg Olofsson K Franzén S Svensson AM Gudbjörnsdottir S. Body mass index as a risk factor for coronary events and mortality in patients with type 1 diabetes. Open Heart. (2018) 5:e000727. doi: 10.1136/openhrt-2017-000727, PMID: 29387430 PMC5786904

[24] Corbin KD Driscoll KA Pratley RE Egan AM Seaquist ER Bergenstal RM. Obesity in type 1 diabetes: pathophysiology, clinical impact, and mechanisms. Endocr. Rev. (2018) 39:629–63. doi: 10.1210/er.2017-00191, PMID: 30060120

[25] Okubo Y Shimamoto M Watanabe K Oshima Y. A case of anti-GAD antibody-positive fulminant type 1 diabetes with severe obesity. J. Japan Diabetes Soc. (2022) 65:444–50. doi: 10.11213/tonyobyo.65.444

[26] Christou MA Christou PA Katsarou DN Papatheodorou E Papanas N Tentolouris N. Effect of body weight on glycaemic indices in people with type 1 diabetes using continuous glucose monitoring. J. Clin. Med. (2024) 13:5303. doi: 10.3390/jcm13175303, PMID: 39274516 PMC11395955

[27] Dubé MC Joanisse DR Prud'homme D Hancox G White MD Tremblay A. Muscle adiposity and body fat distribution in type 1 and type 2 diabetes varying relationships according to diabetes type. Int. J. Obes. (Lond). (2006) 30:1721–8. doi: 10.1038/sj.ijo.0803337, PMID: 16652137

[28] Salle L Julla JB Fagherazzi G Pinto G Renuy A Amadou C. Beyond overweight visceral adiposity is associated with estimation of cardiovascular risk in patients living with type 1 diabetes findings from the SFDT1 cohort. Cardiovasc. Diabetol. (2025) 24:256. doi: 10.1186/s12933-025-02789-3, PMID: 40517273 PMC12166620

[29] Lim S Meigs JB. Links between ectopic fat and vascular disease in humans. Arterioscler. Thromb. Vasc. Biol. (2014) 34:1820–6. doi: 10.1161/ATVBAHA.114.303035, PMID: 25035342 PMC4140970

[30] Neeland IJ Ross R Després JP Matsuzawa Y Yamashita S Shai I. Visceral and ectopic fat atherosclerosis and cardiometabolic disease a position statement. Lancet Diabetes Endocrinol. (2019) 7:715–25. doi: 10.1016/S2213-8587(19)30084-1, PMID: 31301983

[31] Srikanthan P Karlamangla AS. Muscle mass index as a predictor of longevity in older adults. Am. J. Med. (2014) 127:547–53. doi: 10.1016/j.amjmed.2014.02.007, PMID: 24561114 PMC4035379

[32] Koon Yee Lee G Chun Ming Au P Hoi Yee Li G Wong SY Kwok T Woo J. Sarcopenia and mortality in different clinical conditions a meta analysis. Osteoporos Sarcopenia. (2021) 7:S19–27. doi: 10.1016/j.afos.2021.02.001, PMID: 33997305 PMC8088992

[33] Wang Y Luo D Liu J Song Y Jiang B Jiang H. Low skeletal muscle mass index and all cause mortality risk in adults a systematic review and meta analysis of prospective cohort studies. PloS One. (2023) 18:e0286745. doi: 10.1371/journal.pone.0286745, PMID: 37285331 PMC10246806

[34] Wei S Zhang J Zheng H Liu C Wang Y Chen Y. Association of the appendicular skeletal muscle mass to visceral fat area ratio with cause specific mortality in diabetes. Calcif Tissue Int. (2025) 116:85. doi: 10.1007/s00223-025-01389-3, PMID: 40517189 PMC12167329

[35] Shen Y Li M Wang K Zhang W Liu Y Chen J. Diabetic muscular atrophy molecular mechanisms and promising therapies. Front. Endocrinol. (Lausanne). (2022) 13:917113. doi: 10.3389/fendo.2022.917113, PMID: 35846289 PMC9279556

[36] Pollakova D Tubili C Di Folco U De Giuseppe R Battino M Giampieri F. Muscular involvement in long term type 1 diabetes does it represent an underestimated complication. Nutrition. (2023) 112:112060. doi: 10.1016/j.nut.2023.112060, PMID: 37267657

[37] Shimura K Miura J Shen Z Yamaguchi Y Sasaki H Takahashi T. Prevalence and risk factors for skeletal muscle mass loss in individuals with type 1 diabetes. Diabetol. Int. (2024) 16:92–9. doi: 10.1007/s13340-024-00770-1, PMID: 39877446 PMC11769926

[38] Fenner-Pena N Fajardo VC Froes L Carvalho PAM Comim FV Sahade V. Phase angle and body composition in long-term type 1 diabetes in adults: a comparative study in a Brazilian public reference outpatient clinic. Diabetol. Metab. Syndr. (2024) 16:269. doi: 10.1186/s13098-024-01485-8, PMID: 39533433 PMC11559135

[39] An HJ Tizaoui K Terrazzino S Cargnin S Lee KH Nam SW. Sarcopenia in autoimmune and rheumatic diseases: a comprehensive review. Int. J. Mol. Sci. (2020) 21:5678. doi: 10.3390/ijms21165678, PMID: 32784808 PMC7461030

[40] Liu C Wong PY Chung YL Zhang H Lee JS Chan R. Deciphering the "obesity paradox" in the elderly a systematic review and meta analysis of sarcopenic obesity. Obes. Rev. (2023) 24:e13534. doi: 10.1111/obr.13534, PMID: 36443946

[41] Tuttle KR Reynolds CL Kornowske LM Jones CR Alicic RZ Daratha KB. Prevalence and severity of chronic kidney disease in a population with type 1 diabetes from a United States health system: a real-world cohort study. Lancet Reg. Health Am. (2025) 47:101130. doi: 10.1016/j.lana.2025.101130, PMID: 40486991 PMC12145773

[42] Wallace AS Chang AR Shin JI Reider J Echouffo-Tcheugui JB Grams ME. Obesity and chronic kidney disease in US adults with type 1 and type 2 diabetes mellitus. J. Clin. Endocrinol. Metab. (2022) 107:1247–56. doi: 10.1210/clinem/dgab927, PMID: 35080610 PMC9016431

[43] Andersen H Gadeberg PC Brock B Jakobsen J. Muscular atrophy in diabetic neuropathy a stereological magnetic resonance imaging study. Diabetologia. (1997) 40:1062–9. doi: 10.1007/s001250050788, PMID: 9300243

[44] Espino Gonzalez E Dalbram E Mounier R Nguyen Q Lee S Patel R. Impaired skeletal muscle regeneration in diabetes from cellular and molecular mechanisms to novel treatments. Cell Metab. (2024) 36:1204–36. doi: 10.1016/j.cmet.2024.02.014, PMID: 38490209

[45] Koppe L Fouque D Kalantar Zadeh K. Kidney cachexia or protein energy wasting in chronic kidney disease facts and numbers. J. Cachexia Sarcopenia Muscle. (2019) 10:479–84. doi: 10.1002/jcsm.12421, PMID: 30977979 PMC6596400

[46] Carretero JM Rodríguez L García González R Quam RM Arsuaga JL. Exploring bone volume and skeletal weight in the Middle Pleistocene humans from the Sima de los Huesos site (Sierra de Atapuerca Spain). J. Anat. (2018) 233:740–54. doi: 10.1111/joa.12886, PMID: 30280382 PMC6231173

[47] Lloyd JT Alley DE Hochberg MC Shardell M Walston JD Ferrucci L. Changes in bone mineral density over time by body mass index in the Health ABC study. Osteoporos Int. (2016) 27:2109–16. doi: 10.1007/s00198-016-3506-x, PMID: 26856584 PMC5892439

[48] Walowski CO Herpich C Enderle J Braun W Both M Hasler M. Determinants of bone mass in older adults with normal- and overweight derived from the crosstalk with muscle and adipose tissue. Sci. Rep. (2023) 13:5030. doi: 10.1038/s41598-023-31642-4, PMID: 36977715 PMC10050471

[49] Aversa Z Costelli P Muscaritoli M. Cancer induced muscle wasting latest findings in prevention and treatment. Ther. Adv. Med. Oncol. (2017) 9:369–82. doi: 10.1177/1758834017698643, PMID: 28529552 PMC5424865

